# Interventions to mitigate infant food insecurity in high-income countries: an overview of current evidence

**DOI:** 10.1186/s41110-025-00343-5

**Published:** 2025-05-22

**Authors:** Kusum Singal, Flora Douglas, Phil Mackie, Shantini Paranjothy, Miriam Brazzelli

**Affiliations:** 1 Aberdeen Centre for Evaluation, Institute of Applied Health Sciences, University of Aberdeen, Aberdeen, UK; 2 School of Nursing, Midwifery and Paramedic Practice, Robert Gordon University, Aberdeen, UK; 3 Public Health, NHS Grampian, Aberdeen, UK

**Keywords:** Infant food insecurity, Scoping review, Food insecurity interventions

## Abstract

**Aim:**

Infant food insecurity (IFI) is a critical and often overlooked issue in high-income countries. This scoping review aims to identify and summarise interventions that reduce food insecurity or improve nutrition amongst families with infants in these regions.

**Subject and methods:**

We searched the major electronic databases and websites of relevant UK and international organisations from 2010 to 2023 to identify reports written in English assessing food insecurity affecting infants (aged 0 to 2 years). The findings were presented in tables and summarised narratively.

**Results:**

Out of 6194 records identified, 104 studies were screened, with only two studies meeting the inclusion criteria. Both studies were conducted in the USA. The KIND (Keeping Infants Nourished and Developing) intervention improved preventive care for food-insecure families, increasing lead level test completion rates and well-infant visits, but it did not affect weight-for-length at 9 months. The GWCC (Group Well-Child Care) intervention aimed at promoting responsive feeding amongst low-income caregivers but showed no significant impact on infant growth in the first year. However, caregiver interviews revealed important feeding-related themes.

**Conclusion:**

Evidence on interventions addressing infant food insecurity is limited, with none found in the UK. The KIND and GWCC interventions showed mixed outcomes, improving some aspects of care but not significantly affecting infant growth metrics. These findings highlight the need for further research to develop more effective strategies to address the nutritional needs of vulnerable infants in high-income countries.

**Supplementary Information:**

The online version contains supplementary material available at 10.1186/s41110-025-00343-5.

## Introduction

Food security exists when people have physical, social, and economic access to sufficient, safe, and nutritious food to maintain a healthy and active life. In contrast, food insecurity refers to the limited or uncertain availability of nutritionally adequate and safe foods or the inability to acquire food in socially acceptable ways [1]. Food insecurity is not limited to low-income settings; it also affects high-income countries, despite the presence of advanced welfare and food assistance systems. In these contexts, food insecurity particularly impacts socioeconomically disadvantaged populations [2]. Recent estimates using the Food Insecurity Experience Scale (FIES) indicate that in 2023, approximately 8.7% of individuals in high-income countries (i.e. Northern America and Europe) experienced moderate or severe food insecurity [3]. In the United States, 13.5% of households were reported to face food insecurity at some point during 2023, meaning they struggled to provide sufficient food for all family members due to limited resources [4]. Similarly, a small cross-sectional study in Australia found that 25.9% of surveyed individuals were experiencing severe food insecurity [5]. In the UK, the prevalence of food insecurity is likely to be exacerbated by economic instability and rising living costs, contributing to increased risk for infants and young children [6]. However, comprehensive prevalence statistics on infant food insecurity specifically remain limited. Food insecurity in infants is associated with poor outcomes such as detrimental physical, psychological, and behavioural effects on their development [7]. It may lead to malnutrition and push parents to adopt sub-optimal feeding and weaning practises [8]. Such coping strategies may negatively impact the health and well-being of infants, with negative consequences in the short and long term [9]. The child rights-focused guidance for Local Authorities and Health Boards has recently been updated by UNICEF UK Baby Friendly Initiative, First Steps Nutrition Trust and the National Infant Feeding Network (NIFN) to support relevant teams in their joint efforts to ensure that infants and families at risk of food insecurity are given the most appropriate support to meet their needs, with the goals of maximising short- and long-term health and well-being outcomes and minimising risk [10].

Previous research has examined interventions addressing household food insecurity, but fewer studies have specifically examined community-level initiatives targeting infant food insecurity. In 2019, Holley and Mason reviewed food insecurity interventions in high-income settings, providing an overview of policies and programmes mainly aimed at improving food access [11]. However, they did not specifically focus on interventions targeting infant food insecurity.

Our focus on high-income countries is intentional, as these nations often have well-established food assistance programmes and policies, yet food insecurity persists, particularly amongst socioeconomically disadvantaged groups [2]. Understanding the effectiveness of community-level interventions in these settings can inform policies and programmes that address food insecurity whilst also providing insights that may be adaptable to other contexts. By examining the existing body of knowledge and identifying gaps in research and practise, this review will contribute to the understanding of effective strategies for mitigating the impacts of infant food insecurity in affluent nations.

## Methods

This scoping review was carried out in accordance with the Joanna Briggs Institute (JBI) methodology and reported following the Preferred Reporting Items for Systematic Reviews and Meta-Analysis for Scoping Reviews (PRISMA-ScR) [12, 13]. Details of this review are available in the Open Science Framework (https://osf.io/kcpwr/). This review aims to address the following research question:

*What community-level interventions have been proposed and evaluated to reduce the risk of infant food insecurity and improve infant food security in high-income countries?*

## Inclusion criteria

### Participants

The population of interest included infants aged 0 to 24 months and households with infants experiencing food insecurity. We documented the definitions of food insecurity as reported by the authors of the included studies. Acceptable measures of food insecurity could include validated quantitative tools (e.g. the Household Food Security Survey Module) and qualitative indicators, such as reliance on food assistance programmes, economic constraints affecting infant feeding practises, or caregiver-reported struggles to access food.

### Concept

We focused on evidence that explored or evaluated community-level interventions aimed at reducing or improving infant food nutrition in high-income countries. Eligible interventions aimed at improving food supply included any policy to increase local production of nutritious food for infants, changes in food retailing or marketing practises, urban planning, and land zoning modifications to boost the production or sale of nutritious foods, as well as economic development programmes aimed at establishing grocery stores in areas in underserved areas. Eligible interventions designed to improve access to food included measures to enhance purchasing power or economic resources for food procurement, reduce the price of nutritious infant food, and government cash transfer programmes such as social assistance payments or tax credits. Other relevant interventions such as government nutrition assistance programmes in high-income countries (e.g. Supplemental Nutrition Assistance Program [SNAP], Special Supplemental Nutrition Program for Women, Infants and Children [WIC]) were also deemed suitable for inclusion. Studies were considered eligible for inclusion only if they reported at least one outcome measure specific to infants, such as infant weight, growth, feeding behaviour, nutritional status, or indicators of infant-level food insecurity. Although interventions could also address household-level food insecurity, studies were excluded if they did not include infant-specific outcomes.

### Context

Only studies conducted in high-income countries as per the World Economic Situation and Prospects (WESP) 2023 categorisation were included in the review [14]. The specific focus on high-income countries is justified by the common assumption that food insecurity is less prevalent in these settings. However, it remains an underexplored issue, and growing evidence suggests that vulnerable populations—such as infants and low-income families—face significant challenges [15]. This gap underlines the need for a closer examination of targeted interventions. Studies conducted in low- and middle-income countries were excluded, as well as reports of studies that focus exclusively on household food insecurity without providing information on infant food insecurity.

### Types of sources

This scoping review considered studies of any design published in full between 2010 and 2023 that focused on interventions targeting infant food insecurity in high-income countries. This timeframe was chosen to capture the most recent evidence base and reflect contemporary policy and programme developments. Both quantitative and qualitative reports of infant food insecurity from parents, relative caregivers, or professionals who engaged with them were deemed relevant, provided they met our pre-specified inclusion criteria. Reports had to present primary or secondary outcome measures related to infant food insecurity or infant-specific impacts. Grey literature was also considered; however, opinion papers and reports without infant-level data were excluded.

The following databases were searched MEDLINE (Ovid), EMBASE (Ovid), CINAHL (EBSCO), The Cochrane Library, PsycINFO (ProQuest), the Applied Social Sciences Index and Abstracts (ASSIA), and Web of Science (WoS), covering the period from 2010 to 2023. Some of the electronic databases index both published and unpublished studies (e.g. doctoral dissertations and conference abstracts), allowing for partial capture of unpublished research. In addition, grey literature addressing the research question was searched, and reference lists of identified studies were scrutinised for additional publications. Opinion papers were excluded. Details of the search strategies are provided in the attached Supplementary File.

### Selection of the relevant studies and charting of data

**Fig. 1:**
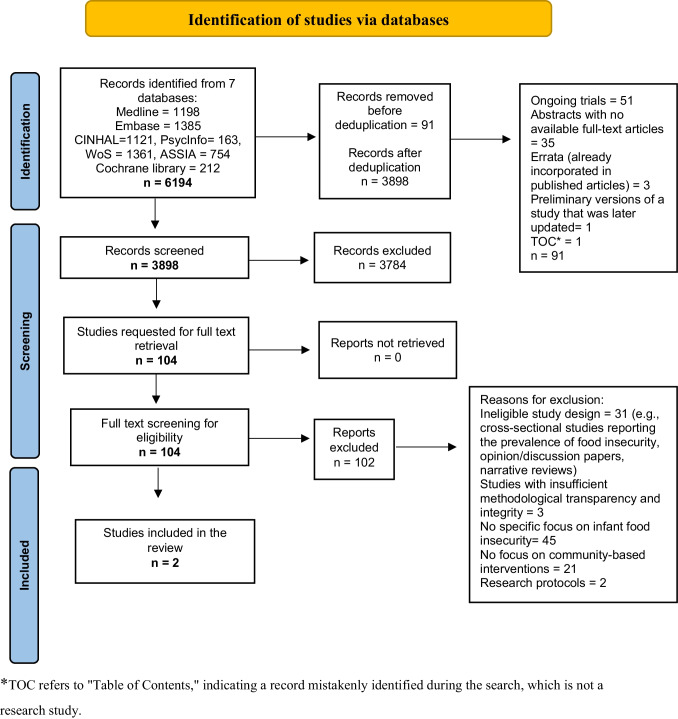
PRISMA diagram showing the study selection process. *TOC refers to “Table of Contents,” indicating a record mistakenly identified during the search, which is not a research study

Results of electronic searches were uploaded in EndNote bibliographic software (version X9 20.3; Clarivate Analytics, PA, USA), and, where possible, duplicate entries were removed. One reviewer conducted the screening of the search results against the pre-specified inclusion criteria. All potentially relevant articles were retrieved in full for content assessment. The selection process is outlined in Fig. 1.

Data extraction was performed by a single reviewer, with a second reviewer verifying the extracted data. From each study, information was collected on study authors, publication year, study design and objectives, target population, study duration, data sources (primary or secondary), data collection methods, characteristics of intervention, and outcome measures (see Table 1). All data were organised using a customised Excel sheet developed for this scoping review.

**Table 1:** Characteristics of the included studies

Study ID	Country	Study design	Aims/objective	Population and measures of food insecurity	Study duration	Primary or secondary source of data and data collection procedure	Type of intervention	Key findings
Beck et al. [16]	USA	Prospective interventional study	To design, implement, refine, and evaluate Keeping Infants Nourished and Developing (KIND), a collaborative intervention focused on food-insecure families with infants	Food-insecure families with infants Food insecurity indicators included reliance on public assistance programmes such as the Supplemental Nutrition Assistance Program (SNAP) and Women, Infants, and Children (WIC)	2 years	The data used in the study includes patient-level demographic, clinical, preventive care, and social risk data. These data points were extracted directly from patient records. Comparisons were made between those who received KIND and those who did not	Supplementary infant formula, educational materials, clinic, and community resources	1042 families with infants received KIND. Recipients were more likely than nonrecipients to have completed a lead level test and developmental screening (both *P* <.001) and received a full set of well-infant visits by 14 months (42.0% versus 28.7%; *P* <.0001) KIND recipients were significantly more likely to have been referred to social work (29.2% versus 17.6%; *P* <.0001) or medical-legal partnerships (14.8% versus 5.7%; *P* <.0001) than no-recipients No significant differences in weight-for-length at 9 months were observed between the two groups
Budge et al. [17]	USA	Prospective interventional study (mixed-methods design)	To evaluate healthy eating through Group Well-Childcare (GWCC) versus individual well-childcare (IWCC). A partnership between the Special Supplemental Nutrition Program for Women, Infants, and Children (WIC) and Yale Pediatric Care Centre in the Northeast United States, largely serving Black and Latino families. The partnership was established to promote responsive feeding practises amongst low-income caregivers, by examining their impact on infant growth and exploring the experiences of caregivers	Low-income caregivers and infants Two-item food insecurity scale was used to identify families at risk of food insecurity	November 2013 and November 2020	Information from routine care appointments for newborns, including physical exams, vaccinations, and anticipatory guidance, was collected directly from families participating in the GWCC and WIC groups	Healthy Eating through GWCC intervention in partnership with WIC	No significant difference in first-year weight-for-length trajectories amongst those in GWCC versus those in IWCC Four major themes around feeding from caregivers’ interviews were identified: *Structural barriers*: Difficulty accessing healthy WIC-approved foods *Conflicting advice*: Caregivers received mixed nutrition advice from various sources, which complicated decision-making *Novel food experimentation*: Caregivers appreciated trying new healthy food, encouraging them to experiment at home. Some caregivers were not keen to try new food because of cultural differences *Responsive feeding adoption*: Many caregivers gained awareness and began adopting responsive feeding practises, although barriers remained for some of them

### Reporting of results

From an initial dataset of 6194 records, 91 duplicates were identified and removed, resulting in a refined dataset of 3898 unique records. After excluding 3784 irrelevant records, we retrieved the full texts of 104 studies for further evaluation. Ultimately, two studies conducted in 2013 and 2023, respectively, met our selection criteria and were included in the final review [16, 17]. Both studies were conducted in the USA, one using a prospective interventional design and the other employing a mixed-method approach.

### Descriptive outcomes

The study by Beck et al. focused on the Keeping Infants Nourished and Developing (KIND) programme, a collaborative initiative to address food insecurity (FI) amongst families with infants living in Cincinnati, Ohio [16]. The programme was developed through a partnership between the Pediatric Primary Care Centre at Cincinnati Children’s Hospital Medical Centre and a foodbank agency (Freestore Foodbank). It was primarily targeted at infants from socioeconomically disadvantaged families. Families identified as food-insecure received infant formula, educational materials on infant nutrition, and referrals to relevant social services, such as social workers, medical-legal partnerships, and public assistance programmes. The results from the first 2 years of the KIND programme show that 1042 families were served, and 1601 cans of formula milk and educational brochures were distributed. Families who participated were more likely to complete preventive care services, including lead testing and developmental screenings compared to those who did not participate. Additionally, they were more frequently connected to social support systems, such as social workers and medical-legal partnerships. The programme was also associated with improved rates of well-infant visits, demonstrating better overall preventive care, especially in terms of monitoring infants’ growth and development. However, it is important to note that the study did not find a statistically significant difference in weight-for-length at 9 months between recipients and non-recipients. Whilst the programme did not lead to significant improvements in infant growth, the broader impact of improved access to healthcare and support services was considered highly valuable. The success of the KIND programme led to its expansion across multiple clinics in the Cincinnati area, showing the potential for scalability and wider impact.

The study by Budge et al. explored a partnership between the Special Supplemental Nutrition Program for Women, Infants, and Children (WIC) and primary care [17]. It aimed to promote responsive feeding practises in low-income caregivers of infants through Group Well-Child Care (GWCC). The study combined quantitative and qualitative methods to assess the programme’s impact on infant growth and caregivers’ experiences. Specifically, infants’ weight-for-length trajectories in group-based care (GWCC) were compared with individual well-childcare (IWCC) before and after the intervention and interviews with caregivers were conducted to gather insights on their experiences with the programme and feeding practises. The results of this study show that no significant improvements were observed in infant weight-for-length trajectories amongst caregivers receiving GWCC compared with those receiving IWCC. Additionally, caregiver interviews revealed four key themes: structural barriers, conflicting advice, novel food experimentation, and adoption of responsive feeding practises (see Table 1 for further details).

## Discussion

Despite the assumption that food insecurity is primarily a concern in low- and middle-income countries, evidence suggests that high-income countries also experience significant food insecurity, particularly amongst low-income and marginalised populations [15]. The present scoping review casts a spotlight on the issue of infant food insecurity within high-income countries, a critical topic that has yet to receive the attention it deserves. By systematically examining the available evidence, it offers a valuable starting point for understanding the landscape of interventions aimed at addressing this problem. The review synthesises existing—though limited—knowledge of the interventions, programmes, and strategies designed to mitigate infant food insecurity in high-income countries. Despite the scarcity of data, it provides valuable insights into a few approaches designed to support vulnerable families with infants in these specific socio-economic settings. The focus on high-income countries underlines the need to understand and tackle infant food insecurity in contexts that are often assumed to be less affected by such a problem. This can be partly attributed to the general perception that food insecurity is less severe or widespread in high-income countries [11]. However, structural inequalities—such as poverty, immigration status, and employment instability—persist in these contexts, creating food access challenges that disproportionately affect families with infants [18]. A key finding of this scoping review is the significant gap in research on interventions aimed at mitigating infant food insecurity in high-income countries. Whilst there is extensive literature evaluating food insecurity amongst adults and older children, the unique vulnerability and needs of infants have been largely overlooked [6].

Several reasons may contribute to the dearth of infant food insecurity initiatives in high-income settings. One important factor is the overlap between breastfeeding recommendations and infancy-specific feeding guidelines, which influences intervention design. Infant-targeted programmes typically place more emphasis on breastfeeding promotion, formula supplementation, and parental feeding education, whereas interventions for older children frequently involve direct food provision (e.g. school meal programmes). Moreover, infant food insecurity is linked to multiple socioeconomic factors such as poverty, parental employment, access to healthcare, and government assistance programmes [8, 19–21]. Effective interventions often require cross-sector collaboration (e.g. healthcare, social services, and food programmes), making evaluation more complicated. Also, there is no universal measurement tool specifically designed to evaluate infant food insecurity, making research challenging.

This scoping review identified two community-level interventions addressing infant food insecurity in high-income countries: the KIND programme [16] and the WIC-Primary Care partnership [17]. Both interventions highlighted the multifaceted nature of food insecurity in infancy, focusing not only on food provision but also on connecting families with essential support systems and improving feeding practises. However, neither study showed significant improvements in infants’ growth. The KIND programme identified food-insecure families and provided targeted support through formula distribution, nutritional education, and referrals to social services. KIND effectively identified not only the healthcare needs of vulnerable families with infants but also broader social and legal challenges, offering valuable resources and support beyond just medical care. In particular, the programme’s success in connecting families with healthcare and legal resources, alongside its expansion, suggests that addressing food insecurity requires a comprehensive, multidisciplinary approach. This aligns with existing literature that stresses the importance of addressing social determinants of health when tackling food insecurity [9]. Whilst no significant improvements in infant growth were observed, the programme improved access to preventive care services, which can indirectly contribute to better health outcomes in the long term. The lack of improvements in infants’ growth may be attributed to the relatively short timeframe of the study or to factors outside the scope of the intervention, such as structural socioeconomic barriers that may persist despite localised support.

Similarly, the WIC-Primary Care partnership focused on promoting responsive feeding practises. Despite the interesting approach, there were no significant improvements in infant growth trajectories. Nevertheless, interviews with caregivers identified key themes that provide valuable insights into the barriers and enablers of effective feeding practises. Structural barriers, such as difficulties accessing healthy foods through WIC, and conflicting nutritional advice highlight the complex social context within which vulnerable families operate. Moreover, the exposure to novel foods and the adoption of responsive feeding practises call attention to the importance of culturally sensitive and practical interventions. As such, these findings illustrate the complexities and challenges caregivers face in implementing recommended feeding practises, emphasising the need for more targeted support to overcome these barriers.

The results of both studies suggest that whilst targeted interventions can improve access to resources and care, they may not directly result in measurable growth outcomes in infants. This could be due to a variety of factors, including the duration of the interventions, the complexity of addressing food insecurity, challenges accessing food, and social determinants including family income and employment status, immigration status, level of education and poverty, which were not fully addressed by the interventions. Moreover, growth trajectories in infants may be influenced by multiple factors, including genetics, nurturing environment, and parental feeding practises, which may not be fully captured through food security interventions alone.

Expanding the work of Holley and Mason [11], this review makes an important contribution to the existing literature by providing the first systematic synthesis of infant food insecurity interventions in high-income nations. Our findings highlight the urgent need for targeted research and policy initiatives to directly address infant food insecurity in these contexts. It also highlights broader socioeconomic factors influencing infant food insecurity, aligning with recent public health studies [18]. However, some limitations must be acknowledged. Despite conducting comprehensive literature searches, we identified only two studies, thereby constraining the breadth of insights and conclusions that can be drawn. Also, the screening of search results by a single reviewer may have introduced potential bias in study selection. Furthermore, the lack of detailed information on intervention strategies in the included studies restricts the assessment of critical components, such as the implementation of responsive feeding in GWCC [17]. These challenges highlight the need for more comprehensive research and robust methodologies in future studies.

## Conclusion

This scoping review provides important insights into community-level interventions aimed at addressing infant food insecurity in high-income countries. Both the KIND programme and the WIC-Primary Care partnership demonstrated valuable improvements in connecting families with resources and addressing broader social and legal challenges. However, neither intervention resulted in significant improvements in infant growth, suggesting that addressing food insecurity requires a multifaceted and sustained approach. Given the limited literature on effective interventions for food-insecure families with infants living in high-income countries, it is crucial to conduct future research to address this gap. Future interventions should consider longer-term evaluations, broader structural solutions, and the integration of culturally appropriate strategies to mitigate infant food insecurity, improve feeding practises, and enhance their impact on infant health and development. Our findings also highlight the importance of combining direct nutritional support with broader social interventions to maximise outcomes for food-insecure families.

## Supplementary Information

Below is the link to the electronic supplementary material.

Supplementary file1 (DOCX 21 KB)

## Data Availability

No datasets were generated or analysed during the current study.
