# Supplementary material for: Interventions to mitigate infant food insecurity in high-income countries: an overview of current evidence
Source: Nutrire. 2025 May 22;50(1):37. doi: 10.1186/s41110-025-00343-5 (PMC12098398; doi:10.1186/s41110-025-00343-5)
Supplement: Supplementary file 1 — Supplementary file1 (DOCX 21 KB) [file 41110_2025_343_MOESM1_ESM.docx]

**Supplementary file**

**Search strategies**

Ovid **MEDLINE**(R) and Epub Ahead of Print, In-Process, In-Data-Review & Other Non-Indexed Citations, Daily and Versions <1946 to May 16, 2023>

1 Infant/ or Infant Care/ or exp Infant Food/ or Breast Feeding/ or Infant Health/ or Infant Welfare/ or infant?.tw,kw. 1105967

2 Food Security/ or exp Food Insecurity/ or exp Food Assistance/ or hunger/ 9556

3 (food adj3 (security or insecurity or supply or assistance or sufficien* or insufficien* or quality)).tw,kw. 32673

4 2 or 3 39408

5 1 and 4 2408

6 afghanistan/ or africa/ or africa, northern/ or africa, central/ or africa, eastern/ or "africa south of the sahara"/ or africa, southern/ or africa, western/ or albania/ or algeria/ or andorra/ or angola/ or "antigua and barbuda"/ or argentina/ or armenia/ or azerbaijan/ or bahamas/ or bahrain/ or bangladesh/ or barbados/ or belize/ or benin/ or bhutan/ or bolivia/ or borneo/ or "bosnia and herzegovina"/ or botswana/ or brazil/ or brunei/ or bulgaria/ or burkina faso/ or burundi/ or cabo verde/ or cambodia/ or cameroon/ or central african republic/ or chad/ or exp china/ or comoros/ or congo/ or cote d'ivoire/ or croatia/ or cuba/ or "democratic republic of the congo"/ or cyprus/ or djibouti/ or dominica/ or dominican republic/ or ecuador/ or egypt/ or el salvador/ or equatorial guinea/ or eritrea/ or eswatini/ or ethiopia/ or fiji/ or gabon/ or gambia/ or "georgia (republic)"/ or ghana/ or grenada/ or guatemala/ or guinea/ or guinea-bissau/ or guyana/ or haiti/ or honduras/ or independent state of samoa/ or exp india/ or indian ocean islands/ or indochina/ or indonesia/ or iran/ or iraq/ or jamaica/ or jordan/ or kazakhstan/ or kenya/ or kosovo/ or kuwait/ or kyrgyzstan/ or laos/ or lebanon/ or liechtenstein/ or lesotho/ or liberia/ or libya/ or madagascar/ or malaysia/ or malawi/ or mali/ or malta/ or mauritania/ or mauritius/ or mekong valley/ or melanesia/ or micronesia/ or monaco/ or mongolia/ or montenegro/ or morocco/ or mozambique/ or myanmar/ or namibia/ or nepal/ or nicaragua/ or niger/ or nigeria/ or oman/ or pakistan/ or palau/ or exp panama/ or papua new guinea/ or paraguay/ or peru/ or philippines/ or qatar/ or "republic of belarus"/ or "republic of north macedonia"/ or romania/ or exp russia/ or rwanda/ or "saint kitts and nevis"/ or saint lucia/ or "saint vincent and the grenadines"/ or "sao tome and principe"/ or saudi arabia/ or serbia/ or sierra leone/ or senegal/ or seychelles/ or singapore/ or somalia/ or south africa/ or south sudan/ or sri lanka/ or sudan/ or suriname/ or syria/ or taiwan/ or tajikistan/ or tanzania/ or thailand/ or timor-leste/ or togo/ or tonga/ or "trinidad and tobago"/ or tunisia/ or turkmenistan/ or uganda/ or ukraine/ or united arab emirates/ or uruguay/ or uzbekistan/ or vanuatu/ or venezuela/ or vietnam/ or west indies/ or yemen/ or zambia/ or zimbabwe/ 1287058

7 "Organisation for Economic Co-Operation and Development"/ 535

8 australasia/ or exp australia/ or austria/ or baltic states/ or belgium/ or exp canada/ or chile/ or colombia/ or costa rica/ or czech republic/ or exp denmark/ or estonia/ or europe/ or finland/ or exp france/ or exp germany/ or greece/ or hungary/ or iceland/ or ireland/ or israel/ or exp italy/ or exp japan/ or korea/ or latvia/ or lithuania/ or luxembourg/ or mexico/ or netherlands/ or new zealand/ or north america/ or exp norway/ or poland/ or portugal/ or exp "republic of korea"/ or "scandinavian and nordic countries"/ or slovakia/ or slovenia/ or spain/ or sweden/ or switzerland/ or turkey/ or exp united kingdom/ or exp united states/ 3483790

9 European Union/ 17637

10 Developed Countries/ 21342

11 7 or 8 or 9 or 10 3499678

12 6 not 11 1197761

13 5 not 12 1657

14 limit 13 to yr="2010 -Current" 1198

**Embase** <1974 to 2023 Week 19>

1 infant/ or infant care/ or exp baby food/ or breast feeding/ or child health/ or infant welfare/ or infant?.tw,kw. 999749

2 food security/ or exp food insecurity/ or food assistance/ 15950

3 (food adj3 (security or insecurity or supply or assistance or sufficien* or insufficien* or quality)).tw,kw. 35183

4 2 or 3 40359

5 1 and 4 2929

6 afghanistan/ or africa/ or africa, northern/ or africa, central/ or africa, eastern/ or "africa south of the sahara"/ or africa, southern/ or africa, western/ or albania/ or algeria/ or andorra/ or angola/ or "antigua and barbuda"/ or argentina/ or armenia/ or azerbaijan/ or bahamas/ or bahrain/ or bangladesh/ or barbados/ or belize/ or benin/ or bhutan/ or bolivia/ or borneo/ or "bosnia and herzegovina"/ or botswana/ or brazil/ or brunei/ or bulgaria/ or burkina faso/ or burundi/ or cabo verde/ or cambodia/ or cameroon/ or central african republic/ or chad/ or exp china/ or comoros/ or congo/ or cote d'ivoire/ or croatia/ or cuba/ or "democratic republic of the congo"/ or cyprus/ or djibouti/ or dominica/ or dominican republic/ or ecuador/ or egypt/ or el salvador/ or equatorial guinea/ or eritrea/ or eswatini/ or ethiopia/ or fiji/ or gabon/ or gambia/ or "georgia (republic)"/ or ghana/ or grenada/ or guatemala/ or guinea/ or guinea-bissau/ or guyana/ or haiti/ or honduras/ or independent state of samoa/ or exp india/ or indian ocean islands/ or indochina/ or indonesia/ or iran/ or iraq/ or jamaica/ or jordan/ or kazakhstan/ or kenya/ or kosovo/ or kuwait/ or kyrgyzstan/ or laos/ or lebanon/ or liechtenstein/ or lesotho/ or liberia/ or libya/ or madagascar/ or malaysia/ or malawi/ or mali/ or malta/ or mauritania/ or mauritius/ or mekong valley/ or melanesia/ or micronesia/ or monaco/ or mongolia/ or montenegro/ or morocco/ or mozambique/ or myanmar/ or namibia/ or nepal/ or nicaragua/ or niger/ or nigeria/ or oman/ or pakistan/ or palau/ or exp panama/ or papua new guinea/ or paraguay/ or peru/ or philippines/ or qatar/ or "republic of belarus"/ or "republic of north macedonia"/ or romania/ or exp russia/ or rwanda/ or "saint kitts and nevis"/ or saint lucia/ or "saint vincent and the grenadines"/ or "sao tome and principe"/ or saudi arabia/ or serbia/ or sierra leone/ or senegal/ or seychelles/ or singapore/ or somalia/ or south africa/ or south sudan/ or sri lanka/ or sudan/ or suriname/ or syria/ or taiwan/ or tajikistan/ or tanzania/ or thailand/ or timor-leste/ or togo/ or tonga/ or "trinidad and tobago"/ or tunisia/ or turkmenistan/ or uganda/ or ukraine/ or united arab emirates/ or uruguay/ or uzbekistan/ or vanuatu/ or venezuela/ or vietnam/ or west indies/ or yemen/ or zambia/ or zimbabwe/ 1670201

7 "Organisation for Economic Co-Operation and Development"/ 2735

8 australasia/ or exp australia/ or austria/ or baltic states/ or belgium/ or exp canada/ or chile/ or colombia/ or costa rica/ or czech republic/ or exp denmark/ or estonia/ or europe/ or finland/ or exp france/ or exp germany/ or greece/ or hungary/ or iceland/ or ireland/ or israel/ or exp italy/ or exp japan/ or korea/ or latvia/ or lithuania/ or luxembourg/ or mexico/ or netherlands/ or new zealand/ or north america/ or exp norway/ or poland/ or portugal/ or exp "republic of korea"/ or "scandinavian and nordic countries"/ or slovakia/ or slovenia/ or spain/ or sweden/ or switzerland/ or turkey/ or exp united kingdom/ or exp united states/ 3717394

9 European Union/ 31188

10 Developed Countries/ 34228

11 7 or 8 or 9 or 10 3750086

12 6 not 11 1521490

13 5 not 12 1936

14 limit 13 to yr="2010 -Current" 1626

15 conference abstract.pt. 4754291

16 14 and 15 241

17 14 not 15 1385

APA **PsycInfo** <2002 to May Week 2 2023>

1 Infant Development/ or Child Care/ or breast feeding/ or infant?.tw,id. 59702

2 food insecurity/ 1397

3 (food adj3 (security or insecurity or supply or assistance or sufficien* or insufficien* or quality)).tw,id. 4522

4 2 or 3 4541

5 1 and 4 193

6 ((low or middle) adj3 countr*).tw. 8249

7 5 not 6 186

8 limit 7 to yr="2010 -Current" 163

**CINAHL**

S1 (MH "Infant") OR (MH "Infant Care") OR (MH "Infant Food") OR (MH "Breast Feeding") OR (MH "Child Welfare") OR TX infant? 377,672

S2 (MH "Food Security+") OR (MH "Food Assistance") 6,285

S3 TX food N3 (security OR insecurity OR supply OR assistance OR sufficien* OR insufficien* OR quality) 20,522

S4 TX S2 OR S3 20,647

S5 S1 AND S4 2,085

S6 (MH "Asia+") OR (MH "South America+") OR (MH "Africa+") OR (MH "Developing Countries") 513,564

S7 S5 NOT S6 1,361

S8 S5 NOT S6 Limiters - Published Date: 20100101-20231231 1,121

**Web of Science** Core Collection

1: infant$ (Topic) Results: 480730

2: food Near/3 (security or insecurity or supply or assistance or sufficien* or insufficien* or quality) (Topic) Results: 108337

3: #1 AND #2 and 2010 or 2011 or 2012 or 2013 or 2014 or 2015 or 2016 or 2017 or 2018 or 2019 or 2020 or 2021 or 2022 or 2023 (Publication Years) Results: 1361

**Cochrane Library**

ID Search Hits

#1 MeSH descriptor: [Infant] this term only 28538

#2 MeSH descriptor: [Infant Care] this term only 582

#3 MeSH descriptor: [Infant Food] explode all trees 1712

#4 MeSH descriptor: [Breast Feeding] this term only 2626

#5 MeSH descriptor: [Infant Health] this term only 84

#6 MeSH descriptor: [Infant Welfare] this term only 91

#7 infant? 73007

#8 #1 or #2 or #3 or #4 or #5 or #6 or #7 73402

#9 MeSH descriptor: [Food Security] this term only 37

#10 MeSH descriptor: [Food Insecurity] explode all trees 58

#11 MeSH descriptor: [Food Assistance] explode all trees 128

#12 food near/3 (security or insecurity or supply or assistance or sufficien* or insufficien* or quality) 2142

#13 #9 or #10 or #11 or #12 2142

#14 #8 and #13 386

#15 MeSH descriptor: [Africa] explode all trees 11348

#16 MeSH descriptor: [Asia] explode all trees 33244

#17 MeSH descriptor: [South America] explode all trees 3860

#18 MeSH descriptor: [Developing Countries] this term only 1139

#19 (low or middle) near/2 countr* 4854

#20 #15 or #16 or #17 or #18 or #19 51633

#21 #14 not #20 227

Limit to 2010-23 212 (CDSR: 5; CENTRAL: 207)

**ASSIA**

S1 infant? 46465

S2 food N/3 (security or insecurity or supply or assistance or sufficien* or insufficien* or quality) 6873

S3 S1 and s2 1205

S4 (S1 and s2) AND pd(20100101-20231231) 828

S5 (S1 and s2) AND (stype.exact("Scholarly Journals") AND pd(20100101-20231231)) 754
